# Supplementary material for: Modeling glioblastoma heterogeneity as a dynamic network of cell states
Source: Mol Syst Biol. 2021 Sep 16;17(9):e10105. doi: 10.15252/msb.202010105 (PMC8444284; doi:10.15252/msb.202010105)
Supplement: Supplementary file 6 — Source Data for Figure 5 [file MSB-17-e10105-s004.zip › Figure5A_sourcedata/GSEA_3017/hallmarks_stateE.GseaPreranked.1621934677158/HALLMARK_ESTROGEN_RESPONSE_LATE.html]

Details for gene set HALLMARK\_ESTROGEN\_RESPONSE\_LATE[GSEA]

|  || Dataset | state63017 |
| Phenotype | NoPhenotypeAvailable |
| Upregulated in class | na\_neg |
| GeneSet | HALLMARK\_ESTROGEN\_RESPONSE\_LATE |
| Enrichment Score (ES) | -0.24132267 |
| Normalized Enrichment Score (NES) | -0.9360415 |
| Nominal p-value | 0.5320262 |
| FDR q-value | 0.63202506 |
| FWER p-Value | 1.0 |
Table: GSEA Results Summary

  

Fig 1: Enrichment plot: HALLMARK\_ESTROGEN\_RESPONSE\_LATE      
 Profile of the Running ES Score & Positions of GeneSet Members on the Rank Ordered List

  

| PROBE | GENE SYMBOL | GENE\_TITLE | RANK IN GENE LIST | RANK METRIC SCORE | RUNNING ES | CORE ENRICHMENT || 1 | CD44 |  |  | 14 | 0.806 | 0.0585 | Yes |
| 2 | PRSS23 |  |  | 21 | 0.722 | 0.1180 | Yes |
| 3 | RAB31 |  |  | 35 | 0.598 | 0.1586 | Yes |
| 4 | TFPI2 |  |  | 166 | 0.322 | 0.0475 | No |
| 5 | MEST |  |  | 215 | 0.285 | 0.0217 | No |
| 6 | CHPT1 |  |  | 216 | 0.284 | 0.0477 | No |
| 7 | FDFT1 |  |  | 266 | 0.252 | 0.0178 | No |
| 8 | CPE |  |  | 315 | -0.262 | -0.0101 | No |
| 9 | CA12 |  |  | 325 | -0.263 | 0.0042 | No |
| 10 | PPIF |  |  | 442 | -0.297 | -0.0940 | No |
| 11 | KIF20A |  |  | 523 | -0.321 | -0.1512 | No |
| 12 | FKBP5 |  |  | 536 | -0.328 | -0.1342 | No |
| 13 | PLK4 |  |  | 551 | -0.334 | -0.1187 | No |
| 14 | BTG3 |  |  | 615 | -0.365 | -0.1535 | No |
| 15 | RNASEH2A |  |  | 633 | -0.378 | -0.1374 | No |
| 16 | GINS2 |  |  | 658 | -0.399 | -0.1268 | No |
| 17 | STIL |  |  | 692 | -0.425 | -0.1236 | No |
| 18 | RBBP8 |  |  | 714 | -0.440 | -0.1061 | No |
| 19 | CDC20 |  |  | 793 | -0.562 | -0.1390 | No |
| 20 | CDC6 |  |  | 807 | -0.584 | -0.0997 | No |
| 21 | FABP5 |  |  | 939 | -1.294 | -0.1231 | No |
| 22 | TOP2A |  |  | 942 | -1.417 | 0.0043 | No |
Table: GSEA details [plain text format]

  

Fig 2: HALLMARK\_ESTROGEN\_RESPONSE\_LATE: Random ES distribution      
 Gene set null distribution of ES for **HALLMARK\_ESTROGEN\_RESPONSE\_LATE**
